# The International Society for Computational Biology 10th Anniversary

**DOI:** 10.1371/journal.pcbi.0030135

**Published:** 2007-06-29

**Authors:** Lawrence Hunter, Russ B Altman, Philip E Bourne

## Introduction

*PLoS Computational Biology* is the official journal of the International Society for Computational Biology (ISCB), a partnership that was formed during the Journal's conception in 2005. With ISCB being the only international body representing computational biologists, it made perfect sense for *PLoS Computational Biology* to be closely affiliated. The Society had to take more of a chance than similar societies, choosing to step away from an existing financially beneficial subscription journal to align with an open access publication as a matter of principle. To our knowledge, ISCB was the first major international scientific society to do so.

Now, as *PLoS Computational Biology* reaches its two-year mark, ISCB simultaneously celebrates its tenth anniversary, having formed officially on June 18, 1997. We early presidents of ISCB reflect on the state of computational biology ten years ago, how far we have come since, and what thought-provoking future challenges might lie ahead with regard to innovations in publishing technologies.

## The State of Computational Biology at the Founding of ISCB

It's hard to imagine how much the computational bioscience world has changed in just ten years. In 1996, there was no journal that had the word “bioinformatics” in its name, GenBank contained fewer than 200,000 DNA sequences (core genomic DNA/RNA, excluding mitochondria, ESTs, etc.), and the National Institutes of Health (NIH) had yet to fund any institutional training programs in bioinformatics or computational biology. By 1996, however, high throughput molecular biology and the attendant need for informatics had clearly arrived. In addition to the founding of ISCB, 1996 saw the sequencing of the first genome of a free-living organism (yeast) and Affymetrix's release of its first commercial DNA chip.

The hot topics in bioinformatics circa 1996, at least as reflected by the conference on Intelligent Systems for Molecular Biology (ISMB) at Washington University in St. Louis, included issues which have largely been solved (such as gene finding or sequence assembly), as well as ones which have proven richer than all expectations (such as ontological foundations for knowledge models). Although not yet as global as the current Society, attendees in 1996 came from Canada, Denmark, Germany, Japan, the United Kingdom, and the United States. ISMB '96 attendees also included all of the presidents of ISCB so far.

By the time the Society was founded, bioinformatics was gaining notice in the broader scientific community. In June of 1996, *Science* published a “News and Comment” piece entitled “Hot Property: Biologists Who Compute” suggesting that competition among drug companies and other industrial concerns for the relatively few people skilled in bioinformatics was so intense that universities would not be able to attract enough faculty to teach the field to new students. While perhaps not quite as dire as all that, in 1996 there were fewer than half a dozen training programs that offered a Ph.D. in bioinformatics or computational biology.

## ISCB Conferences: Join the Leaders in Your Field

More than most scientific societies, ISCB is closely tied to its conferences. A significant part of the original motivation for founding the Society was to provide a stable financial home for the ISMB conference. The first few conferences were sufficiently successful to create a financial nest egg that was used each year to start the process of planning and executing the next year's meeting. Initially, relatively modest checks were cut and sent from one organizer to another informally. As the size of these checks increased, the organizers (and their home institutions) became increasingly uncomfortable exchanging them informally, and decided that they needed an organization—thus, ISCB.

The basic rules and goals for ISCB were hashed out in an unforgettable late-night dinner on the beaches of Halkidiki, Greece, at ISMB '97. Not surprisingly, the idea of an organization brought much more than the convenience of a bank account for the conferences. There were formative discussions about advocacy, education, travel support, and other activities. However, the role of conferences remained central. It was clear that the ISMB meeting was the primary “product” of the new society. That dinner also paved the way for the current relationship between ISCB and two of the other premier meetings in computational biology: Research in Computational Molecular Biology (RECOMB) and the Pacific Symposium on Biocomputing (PSB), many of whose organizers were present on that Greek beach.

In subsequent years, the Society has formed alliances with other conferences as well, most notably the European Conference on Computational Biology (ECCB) and the Asia Pacific Bioinformatics Network's International Conference on Bioinformatics (InCoB). When ISMB is held in Europe, as it will be this summer, ISMB and ECCB are held jointly. More recently, ISCB has begun organizing smaller regional or specialty meetings such as the Rocky Mountain Bioinformatics Conference.

Merging the conference cultures of molecular biology (where conferences provide an unpublished way to share recent research results and speakers are largely invited) and computer science (where conferences are the primary publication venue for new results, and speaking slots are based on peer-reviewed submissions) has not always been easy. Today's meetings are a remarkable blend that offers a snapshot of the latest, most important results, is published in Medline-indexed proceedings, and balances invited and reviewed talks.

This really makes perfect sense—bioinformatics and computational biology are fundamentally collaborative, interdisciplinary fields where high-bandwidth two-way communication is critical. Of course, such a group of scientists would base their professional society on opportunities to meet in person, interact, tutor, and present work! Our financial dependence on meetings is also rational in the face of our relationship to PLoS (Public Library of Science). To rest primarily on income from journals, as most scientific societies do these days, appears to trade short-term windfalls for long-term uncertainty, not to mention failing to address the important issues of open access and dissemination. To rest our Society's finances more on the health of its meetings may turn out to be a more robust strategy. When funding is good, the meetings flourish and attract many newcomers and the curious, allowing us to build a nest egg. Even when times are hard, meetings provide a critical lifeline for essential scientific communication and are difficult for practicing scientists to skip. A high-quality meeting is much more likely to yield value to the individual scientist in terms of ideas, scouting the competition, and offering collaborative opportunities than a personal journal subscription.

ISCB's conference strategy also aims at creating a truly global community for bioinformatics and computational biology. ISCB conference venues have included not only North American and European sites, but also Hawaii, Australia, Japan, and, most recently, Brazil. The policies of moving the conference around the world in a regular, judicious manner have allowed the field to promote its importance and vitality in multiple venues. There is sometimes a cost in terms of total attendance figures. These must certainly be strategically considered in light of our financial dependence on meetings, but the benefits of engaging new regions and new groups of scientists are quite significant.

Finally, the ISCB conferences play an important role with respect to publication. More than any other biological meeting, ISCB conference proceedings are an important part of the archival literature in bioinformatics and computational biology. These proceedings are peer-reviewed at a level rivaling some journals, and some are indexed in Medline and PubMed. References to these often seminal papers frequently appear in more traditional archival journals, and this should be a point of pride. Our conferences are previewing work that is of high and lasting impact. *PLoS Computational Biology* contains several examples of work that was first presented at an ISCB conference in the form of an oral abstract, and then published in expanded form in the Journal. PLoS is also experimenting with the publication (in revised form) of tutorials presented at the meetings as well. This is a two-way relationship: the PLoS Track at ISMB facilitates presentation of some of the most important work published in the Journal in the previous year and features early results of Journal articles yet to be published. We believe that the unique relationships among the Society, its conference, and *PLoS Computational Biology* are an important strength for all of us, and invite you to ISMB/ECCB 2007 in Vienna to see this synergy first hand.

## ISCB and *PLoS Computational Biology*: Leaders in Open Access Publishing

ISCB has changed a great deal in the ten years since it began—but so has scientific publishing. Societies and journals are often intimately linked, as the American Chemical Society is with the *Journal of the American Chemical Society,* although of course there are both societies and journals without such links. The current relationship between ISCB and the Public Library of Science through *PLoS Computational Biology* falls somewhere in the middle. Both ISCB and PLoS exist as separate organizations, and they formed a partnership through a letter of agreement. In reality, this means each disseminating the work done by the other. It would be easy to stop there, but the relationship is much more than that. With such a partnership, the Journal becomes a collective voice for the scientific community it represents. *PLoS Computational Biology* should represent the best work and interests of ISCB members.

The need for a relationship between ISCB and journals was recognized early. In 1998, Russ Altman forged a relationship with Oxford University Press (OUP), establishing the journal *Bioinformatics* as the official journal of ISCB. This was very valuable for increasing the visibility of both a fledgling society and a fledgling journal, and had financial incentives as well. The major cost of being a member of ISCB went to OUP in return for a subscription to *Bioinformatics;* royalties went back to ISCB based on overall journal subscription sales. A publications committee was established in 2000 to oversee these activities.

In 2004, when this contract was up for renewal, a very bold initiative was undertaken. With much heated debate, and with a narrow margin, the ISCB Board of Directors chose *PLoS Computational Biology* as the official journal of ISCB. Why the controversy? PLoS is an open access, nonprofit publisher and is not likely to incur profit margins as does a subscription-based publisher, and so the Society does not benefit financially from the arrangement. There is no “income” to ISCB other than one PLoS-sponsored membership for each published research article, and authors must pay to publish their work, although they can request a fee waiver if they don't have access to sufficient funds. The good news is that more and more funding agencies are helping to provide funds to cover open access publishing fees, and, most important of all, anyone can read a paper for free and reuse it without restriction. One could think of this as ISCB contributing to underwriting part of the cost of disseminating our science to the broadest audience.

We former presidents (including Philip E. Bourne, now the Editor-in-Chief of *PLoS Computational Biology*) believe that it is incumbent on the Society to be an “early adopter,” particularly of computational technologies that have the potential to transform the conduct of our science. The Society has long supported “open source” approaches to distribution of scientifically significant computer code (leaving room for other means as well), and the Board of Directors felt that “open access” was similarly important to our field, despite the initial financial foregone income to this far-from-wealthy scientific society.

Being the first among scientific societies to officially adopt an open access journal was a bold move. Ten years hence, it will be fascinating to look back and see what came of this experiment, how the Society weathers the financial storms of middle age, and whether other societies have followed our lead.

The world of scientific publishing will continue on its path of radical change, driven in part by developments in information technology. The members of ISCB are makers and users of those developments, and PLoS is a dynamic organization willing to work with those making change. What can we do together?

Here is a challenge to future ISCB presidents: journals are becoming more like databases, and databases are becoming more like journals; can we not capitalize on that to further scientific comprehension? How a paper's impact is measured (in the future perhaps as a knowledge blob in cyberspace) is changing. Beyond traditional citations, downloads are becoming important, as is how collaborative the work is. How can we capitalize on this to further highlight the work of Society members? Then there is the content as a subject of scientific study. ISCB includes members who are experts in ontologies, semantic content, and data and literature mining; how can these technologies be applied to our own journal to make it more valuable to scientists? Can we bring further recognition to the Journal and ISCB as leaders of innovation in scientific publishing? ISCB has the expertise and the motivation to make this happen, so stay tuned.

 Meanwhile, we are pleased to report that ISCB has reached its 10th anniversary with a commendable list of achievements in conferences, affiliations, partnerships, and services to our ever-growing membership. Likewise, upon concluding its second year of publishing, *PLoS Computational Biology* has grown by leaps and bounds in the areas of submissions, published research articles per issue, and the size and scope of the editorial board. The Journal is also on track to becoming financially self-sustaining, which will demonstrate the viability of the open access model to the publishing community and ensure its permanence among scientific journals. Ten years from now, as ISCB celebrates its 20th anniversary, we look forward to being equally proud of our continued association with *PLoS Computational Biology* as an official journal of ISCB.

